# Nuclear Progesterone Receptor Expressed by the Cortical Thymic Epithelial Cells Dictates Thymus Involution in Murine Pregnancy

**DOI:** 10.3389/fendo.2022.846226

**Published:** 2022-04-14

**Authors:** Soo Hyun Ahn, Sean L. Nguyen, Tae Hoon Kim, Jae-Wook Jeong, Ripla Arora, John P. Lydon, Margaret G. Petroff

**Affiliations:** ^1^ Department of Pathobiology Diagnostic Investigation, College of Veterinary Medicine, Michigan State University, East Lansing, MI, United States; ^2^ Institute for Integrative Toxicology, Michigan State University, East Lansing, MI, United States; ^3^ Cell and Molecular Biology Program, Michigan State University, East Lansing, MI, United States; ^4^ Department of Obstetrics, Gynecology & Reproductive Biology, Michigan State University, Grand Rapids, MI, United States; ^5^ Department of Obstetrics, Gynecology, and Reproductive Biology, Institute for Quantitative Health Science and Engineering, Michigan State University, East Lansing, MI, United States; ^6^ Department of Molecular and Cellular Biology, Baylor College of Medicine, Houston, TX, United States; ^7^ Department of Microbiology and Molecular Genetics, Michigan State University, East Lansing, MI, United States

**Keywords:** thymus, pregnancy, involution, fertility, progesterone receptor (PGR)

## Abstract

Progesterone is a gonadal pro-gestational hormone that is absolutely necessary for the success of pregnancy. Most notable actions of progesterone are observed in the female reproductive organs, the uterus and the ovary. Acting through the nuclear progesterone receptor (PGR), progesterone prepares the endometrium for implantation of the embryo. Interestingly, the maternal thymus also is a known expressor of *Pgr*; its absence is associated with murine pregnancy complications. However, the localization of its expression and its functional importance were not known. Here, we used a transgenic dual fluorescent reporter mouse model and genetic deletion of *Pgr* in Foxn1+ thymic epithelial cells (TEC) to demonstrate TEC-specific *Pgr* expression in pregnancy, especially in the cortex where thymocyte maturation occurs. Using our TEC-specific *Pgr* deletion mouse model, we demonstrate that TEC-specific *Pgr* is necessary for pregnancy-induced thymic involution in pregnancy. Our investigation reveals that PGR expression is upregulated in the cortical thymic epithelial cells during pregnancy, and that PGR expression is important for thymic involution during murine pregnancy.

## Introduction

The thymus is a compartmentalized immune organ dedicated to the maturation of thymocytes into T cells that serve to protect the body from pathogens while maintaining tolerance to self-antigens. After precursor cells migrate to the thymus from the bone marrow, thymic stromal epithelial cells provide sequential developmental signals as T cells migrate through distinct thymic compartments. Thymocytes first encounter cortical thymic epithelial cells (TEC) as they migrate through the cortex for positive selection, which ensures survival of thymocytes with properly arranged surface T cell receptors (TCR). Thymocytes expressing TCR together with the CD4 and CD8 coreceptors then move into the medulla, where they face negative selection, in which they encounter self-antigens expressed by medullary TEC (mTEC). Here, the mature TCR repertoire is defined: T cells reactive to self-antigen undergo cell death or differentiate into regulatory T cells. The latter, as well as those that do not react strongly to the self-antigens expressed by the mTECs, emigrate to the periphery for immunological surveillance (1).

Thymic function is closely tied to immune function: inappropriate T cell development can result in immunodeficiencies and autoimmunity (2, 3). In pregnancy, during which the maternal immune system must tolerate the semi-allogeneic fetus, the maternal thymus involutes, shrinking in size and cellularity to 50% or less of pre-pregnancy values. Kendall and Clarke (4) observed this phenomenon and postulated the involvement of the ovarian hormones progesterone and estradiol, since both rise during pregnancy. Using progesterone receptor (*Pgr*)-deficient mice together with thymic transplantation, Tibbetts et al. (5) demonstrated that progesterone and estradiol can cause thymic involution in a manner dependent on thymic expression of *Pgr*.

Despite these observations, the mechanisms by which progesterone receptor causes thymic involution is poorly understood. Further, pregnancy-associated thymic involution is postulated to contribute to maternal immune tolerance to the fetus (4–6). However, the mechanisms by which thymic involution influences pregnancy outcome is poorly understood. Here, we combined basic experimental approaches, transgenic mouse models, and a TEC-specific *Pgr* knockout model to elucidate how TEC-*Pgr* influences thymus involution and fertility in mice.

## Materials and Methods

### Mice

Experiments with animals complied with NIH’s *Guide for the Care and Use of Laboratory Animals and* were approved by the Institutional Animal Care and Use Committee at Michigan State University. *Pgr^Cre/+^* mice (7) and *Pgr^f/f^* (8) were a gift from Dr. John Lydon (Baylor University) and Dr. Jay Ko (University of Illinois, Urbana Champaign). Breeding pairs of *Rosa^mTmG/mTmG^* (9), *Foxn1* ^Cre/+^ (10), and wild type (WT) C57BL/6J and Balb/cJ were purchased from Jackson Laboratories (Bar Harbor, ME). All animals were on the C57BL/6J background and kept in conventional caging with a 12-hour light/dark cycle and fed *ad libitum*. Female transgenic animals (8-10 weeks old) were mated with 11-20-week-old wild type (WT) C57BL/6J or Balb/c males for fertility studies. Females were sacrificed prior to pregnancy and on day of gestation (GD) 6.5, 14.5, 16.5, and 18.5. The presence of the mucus plug was designated GD0.5.

### RNA Extraction and Real Time-Quantitative Polymerase Chain Reaction

Total RNA was extracted from thymus obtained from C57BL/6J non-pregnant and pregnant (GD16.5) animals to measure transcript levels of genes. After euthanization, thymi were harvested, frozen in liquid nitrogen, and stored at -80°C. RNA was extracted using TRIzol reagent (ThermoFisher, Waltham, MA, USA). Whole thymi were submerged in 1mL of TRIzol and dissociated using Omni Bead Homogenizer (Bead Rupter 12, catalogue number: 19-050A, Omni International Inc., GA, USA). Homogenate was transferred to Phase Lock Gel-Heavy tubes (1.5mL), 0.2mL of chloroform was added per 1mL of TRIzol Reagent, then centrifuged for 10 minutes at 12,000g, with centrifuge set at 4°C. The clear, aqueous phase was transferred to a 1.5mL sterile Eppendorf tube, and RNA was precipitated by adding 0,5mL of isopropyl alcohol per 1mL of TRIzol Reagent. Samples were mixed by inversion, then incubated at room temperature for 10 min, and centrifuged at 12,000g for 10 minutes at 4°C. The RNA pellet was washed with 1mL of 75% ethanol, centrifuged at 7,500g for 5 minutes at 4°C. The resultant RNA pellet was dried at room temperature and dissolved using sterile RNAse free water. RNA quality was assessed using a Nanodrop Lite spectrophotometer (ThermoFisher, Waltham, MA, USA). Quantitect Reverse Transcription Kit (Qiagen, Hilden, Germany) was used for cDNA synthesis from the RNA template followed by real-time quantitative polymerase chain reaction (RT-qPCR) using Taqman Probes (**Table 1**) on a QuantStudio 5 instrument for 35 cycles (Applied Biosystems, Waltham, MA, USA). The Taqman probes are validated by the manufacturer to have amplification efficiencies close to 100%. Fold change was calculated using the delta-delta cycle threshold (ddCT) method normalized to *Gapdh* using a custom-made RStudio package, tidyQ (https://soohyuna.github.io/tidyQ/)

**Table 1:** List of Taqman probes used for RT-qPCR.

Gene name	Protein name	Taqman #
*Tnfrsf11a*	Tumor Necrosis Factor Receptor Superfamily, Member 11a, NFKB Activator	Mm00437129_m1
*Tnfsf11*	TNF Superfamily Member 11	Mm00441906_m1
*Trap1a*	Tumor rejection antigen P815A	Mm00495785_m1
*Prl8a2*	Decidual PRL-related protein	Mm01135453_m1
*Hbb-y*	Fetal hemoglobin	Mm00433936_g1
*Gad1*	Glutamate decarboxylase 1	Mm04207432_g1
*Fezf2*	Fez family zinc finger protein 2	Mm01320619_m1
*Aire*	Autoimmune Regulator	Mm00477461_m1
*Pgr*	Progesterone receptor	Mm00435628_m1
*Pgrmc1*	Membrane progesterone receptor 1	Mm00443985_m1
*Pgrmc2*	Membrane progesterone receptor 2	Mm01283154_m1
*Esr1*	Estrogen receptor 1	Mm00433149_m1
*Esr2*	Estrogen receptor 2	Mm00599821_m1
*Gapdh*	Glyceraldehyde-3-phosphate dehydrogenase	Mm99999915_g1

### Immunofluorescence and Imaris

For immunofluorescence, tissues were fixed in 4% PFA at 4°C, transferred to 30% sucrose overnight, embedded in OCT medium (Sakura Finetek, Torrance, CA), and frozen in a bath of 2-methylbutane chilled in liquid nitrogen. Tissue cryosections (5µm) were prepared and stained using the following antibodies: rabbit monoclonal anti-progesterone receptor (PGR) (SP2, catalogue number: SAB5500165-100UL, tested concentration at 1:400, Sigma-Aldrich, St. Louis, MO); cytokeratin 8 (cTEC marker) (Troma-1, 1mL supernatant, stock concentration: 50ng/mL; tested concentration: 1ng/mL, Developmental Studies Hybridoma Bank, IA, USA), and cytokeratin 5 (mTEC marker) (stock concentration: 1mg/mL; tested concentration: 2ug/mL, Biolegend, San Diego, CA). Goat anti-rabbit IgG-Alexa Fluor 546 and goat anti-rat IgG-Alexa Fluor 488 (stock concentration: 2mg/mL, tested concentration: 0.01mg/mL, ThermoFisher, Waltham, MA, USA) were used as secondary antibodies, and PGR+ areas from stained sections were quantified using CellProfiler (11).

**Figure 1:**
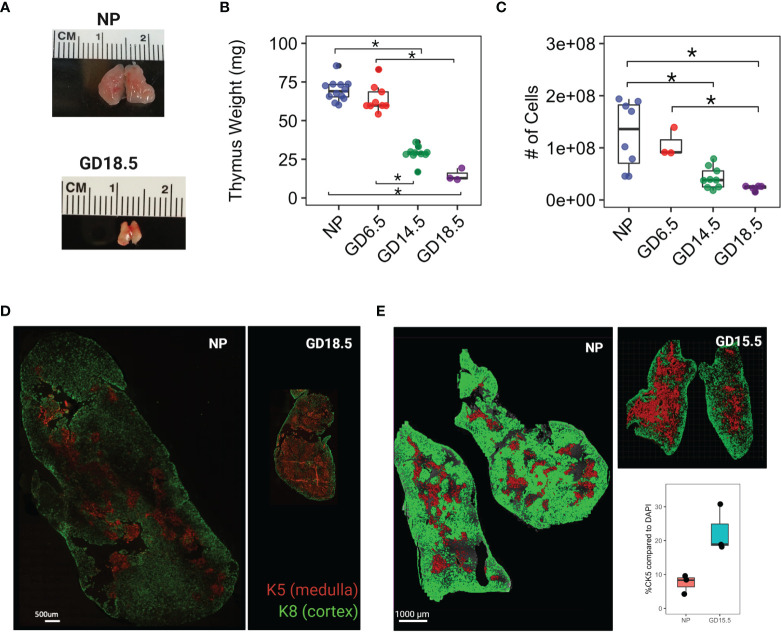
Thymus involution in pregnancy. **(A)** Comparison of gross anatomy of female murine thymus in non pregnant (NP) and GD18.5. **(B)** thymus weight throughout pregnancy. **(C)** Total cell number from thymus throughout pregnancy. **(D)** Immunofluorescence staining of NP and GD18.5 thymus for cytokeratin 5 (K5) and cytokeratin 8 (K8) denoting medulla and cortex, respectively, to illustrate the changes in the volume occupied by each compartment in pregnancy. scale bar = 500μm. **(E)** Comparison of area coverage by medulla (red) to DAPI (entire surface area) in NP and GD15.5 thymus using IMARIS surface rendering function. scale bar = 1000μm. Statistical analysis was performed by non-parametric Kruskal-Wallis test followed by Dunn *post hoc* test. *p < 0.05, Each dot represents one mouse.

To image the entire section of the non-pregnant and pregnant thymus, 5µm thymic sections were first stained with anti-cytokeratin 5 (medulla), anti-cytokeratin 8 (cortex), counterstained with DAPI (Vectashield), and stitched using Leica TCS SP8 X Confocal Laser Scanning Microscope System at 10x magnification. The resulting LIF files were imported into Imaris v9.2.1 (Bitplane). Under surpass mode, surface was created for areas positive for cytokeratin 5, cytokeratin 8 and DAPI using the surface module. Summary statistics were exported from each file and compiled to calculate percent coverage by cytokeratin 5 positive areas in comparison to DAPI (**Figure 1E**).

### Generation of Thymic Epithelial Cell (TEC)-Specific Pgr Knock-Out Mice

Heterozygous *Foxn1^cre/+^* females were crossed to homozygous *Pgr^f/f^* males. Resulting F1 females were crossed to *Pgr^f/f^* males to create experimental *Foxn1^Cre/+^;Pgr^f/f^* (*Pgr^d/d^*) and control *Foxn1^+/+^*;*Pgr^f/f^* (*Pgr^f/f^* or *Pgr^f/+^*) F2 females. To create reporter lines, female *Foxn1^Cre/+^* or *Pgr^Cre/+^* were crossed with dual-fluorescence *Rosa26^mTmG/mTmG^* males to generate *Foxn1^Cre/+^*; *Rosa26^mTmG/+^* and *Pgr^Cre/+^;Rosa26^mTmG/+^* offspring. DNA was extracted from the tail, spleen, ovary, and thymus to genotype for the presence/absence of the floxed Pgr gene. Primer sequences to genotype *Pgr^f/f^* animals were provided by the group of Dr. Jay Ko (University of Illinois at Urbana-Champaign). Three primers were used to generate WT band at 226bp, flox band at 276bp and a KO band at 370bp in length:

- fPR-F: GTATGTTTATGGTCCTAGGAGCTGGG
- fPR-R: TGCTAAAGGTCTCCTCATGTAATTGGG
- fPR-DRp1: GTCCTCCCACTTGCCCCATTCTCAC

Polymerase Chain Reaction (PCR) protocol is as follows: Step 1: 5 minutes at 94°C, Step2: 1 minute at 94°C, Step 3: 1 minute at 57°C, Step 4: 2 minutes at 72°C. Steps 2 to 4 were repeated for 35 cycles, followed by a hold for 10 minutes at 72°C. PCR products were run on a 2% agarose gel and exposed under UV light using a MyECL imager (ThermoFisher, Waltham, MA, USA). As per the description from Jackson Laboratory where *Foxn1^cre^* strain was purchased (stock number: 018448), *Foxn1* is also expressed by the skin keratinocytes. The F1 genotype results show the presence of WT, KO and flox alleles due to the tail digest containing mixture of *Foxn1+* cells, which possess alleles of WT/KO, and the rest, which remain WT/FL. We leveraged this information to identify *Pgr^d/d^* animals using tail snips, genotype of which show both a KO and flox bands from the tail snip, indicating that *Foxn1^cre+^* cells are KO/KO, while rest are FL/KO. This is also true in the ovary, uterus, spleen, and thymus as shown below. Thymus also shows FL/KO because of the presence of thymocytes and other cell types in the thymus that do not express Foxn1, and thus remain FL/KO (**Supplementary Figure 1**).

### Fertility Trial of F2 Females

Female control (*Pgr* ^f/f^) and *Pgr^d/d^* mice (6-8 weeks old) were bred to major histocompatibility complex (MHC)-matched (C57BL/6J) or mismatched (Balb/c) males for 4-6 cycles of continuous pregnancy. Pups were counted and weighed at weaning (21 days postpartum), sexed using ano-genital distance, then euthanized.

### Serum Sex Hormone Measurement

Sera were collected from nonpregnant (NP) and pregnant females at GD16.5 *via* cardiac puncture and assayed for progesterone and estradiol by enzyme-linked immunosorbent assay (ELISA) at the University of Virginia Center for Research in Reproduction Ligand Assay and Analysis Core (Charlottesville, VA, USA). Progesterone ELISA (Catalog no. IB79105, IBL, Minneapolis, MN) had an assay range of 0.3-40 ng/ml, and intra-/inter-assay coefficients of variation of 6.0% and 10.1%, respectively. Estradiol (catalog no. ES-190S-100; Calbiotech, Spring Valley, CA) was measured with an assay range of 3-300 pg/ml and intra-/inter-assay CVs of 5.7% and 10.2%. Further details of assays are available (https://med.virginia.edu/research-in-reproduction/ligand-assay-analysis-core, last accessed June 10, 2021).

### Flow Cytometry

To prepare a single cell suspension of thymocytes, thymus was dissected from the mouse, placed on top of 70 µm mesh, pressed through the filter using a back of 1mL syringe, collected into a 15mL tube, and centrifuged at 400g for 5 min. Single cell suspensions were filtered through 70um mesh, resuspended in flow staining buffer (FSB, 5% fetal bovine serum in PBS) and incubated with ACK (Ammonium-Chloride-Phosphorus) buffer to lyse red blood cells. Cell number was determined using a Countess II (ThermoFisher, Waltham, MA, USA). For viability, all cells were stained with Live/Dead™ Fixable Blue Dead Cell Stain kit (Catalog number: L23105, Thermofisher, Waltham, MA, USA) on ice for 30 min followed by Fc receptor block (Biolegend, San Diego, CA, USA) on ice for 20 min. Prior to cell surface staining, cells were stained with anti-CCR7 antibody at 37°C for 30 min. Post washing in FSB twice, cells were stained using a cocktail of cell-surface antibodies for 30 min on ice. All samples were analyzed using spectral flow cytometry, Cytek^(R)^Aurora (Cytek Biosciences, CA, USA), with 5 laser capability, at Michigan State University Flow Cytometry Core. Flow cytometric analysis software, Kaluza v1.3 (Beckman Coulter, IN, USA) was used for analysis and generation of gating strategy. A full list of the antibodies is available in **Table 2**. The following antibodies were purchased from Biolegend: CD4 (GK1.5), CD8 (53-6.7), CD25 (PC61), CD44 (IM7), CD69 (H1.2F3), and CCR7 (4B12).

**Table 2:** List of antibodies used for flow cytometry.

Antibody	Fluorochrome	Clone	Stock Concentration	Experimental Concentration	Vendor (Cat No.)
CD4	Alexa Fluor 488	GK1.5	0.5mg/mL	1:200 (0.0025mg/mL)	Biolegend (100434)
CD8	Alexa Fluor 700	53-6.7	0.5mg/mL	1:200 (0.0025mg/mL)	Biolegend (100730)
CD25	APC	PC61	0.2mg/mL	1:50 (0.004mg/mL)	Biolegend (102012)
CD44	BV711	IM7	0.1mg/mL	1:200 (0.0005mg/mL)	Biolegend (103043)
Live Dead	Blue			1:1000	ThermoFisher (L23105)

### Statistical Analysis

Power analysis using G*Power was conducted to determine the sample size for all experiments. RStudio software was used to conduct statistical analysis and to graph all data. The Shapiro test was used to assess normality, which was followed by either parametric (normally distributed) or non-parametric (not normally distributed) test. For normally distributed data, Student’s t-test was used to compare the means of two independent samples; one-way ANOVA was used for more three or more independent samples. For non-parametric data, Wilcoxon and Kruskal-Wallis tests were used. In all incidences, *P* values less than 0.05 were deemed statistically significant.

## Results

### Thymic Medullary Islands Re-Distribute Due to Cortical Shrinkage in Pregnancy

We first assessed changes in thymic architecture, mass, and cellularity across pregnancy. C57BL/6J females were paired with C57BL/6J males and sacrificed on GD6.5, 14.5, or 18.5. We confirmed that thymic mass and cellularity decreased progressively across murine pregnancy (Mass: 69.5 ± 6.9 mg vs. 14.7 ± 4.0mg in NP and GD18.5, respectively; Cellularity: 125x10^6^ ± 64x10^6^ vs. 23x10^6^ ± 4.7x10^6^ cells in NP and GD18.5, respectively) (**Figures 1A–C**, *p*<0.05). To compare the changes in the thymic architecture between virgin and pregnant female mice, we performed immunofluorescence on thymic sections with anti-cytokeratin 5 (K5) and anti-cytokeratin 8 (K8) antibodies, which define medullary and cortical thymic epithelial cells, and the areas they occupy, respectively. Using IMARIS software to quantify the areas of the cortex and medulla, we observed shrinkage of cortical area together with relative expansion of medullary area with pregnancy (**Figure 1E**).

### Thymic Expression of *Pgr* Increases With Pregnancy

**Figure 2:**
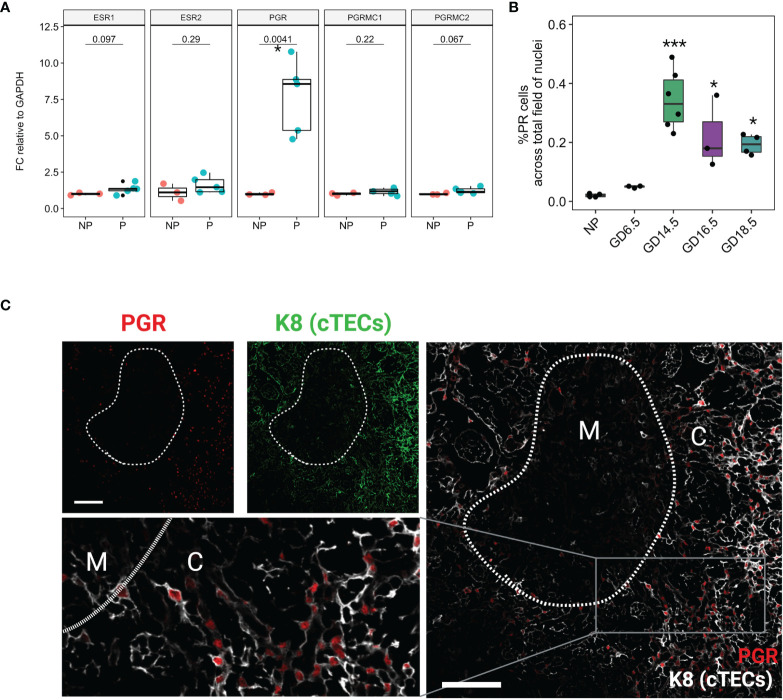
Nuclear Progesterone Receptor expression increases in the thymus in pregnancy. **(A)** RT-qPCR for *Esr1, Esr2, Pgr, Pgrmc1*, and *Pgrmc2* between NP (n=3) and P (GD16.5, n=5) thymus. Each dot represents one mouse. **(B)** % PR positive nuclei calculated from field of DAPI from NP (n=4), GD6.5 (n=3), GD14.5 (n=6), GD16.5 (n=3), and GD18.5 (n=4) thymus; see **Supplementary Figure 1** for representative images. *p < 0.05, ***p < 0.0001; one-way ANOVA with Dunnett’s *post-hoc* test with comparisons to NP controls. Each dot represents one mouse. **(C)** Immunofluorescence staining of GD14.5 thymus with antibodies specific to cytokeratin 8 (K8) and nuclear PGR. C, cortex; M, medulla, Scale bar = 100μm, 200x magnification.

Because estrogen and progesterone play an important role in pregnancy-associated thymic involution, we analyzed the gene expression of steroid hormone receptors in the thymus. We used RT-qPCR to quantify mRNA expression of *Pgr, Esr1, Esr2, Pgrmc1*, and *Pgrmc2* in the thymus of NP and GD16.5 C57BL/6J females. *Pgr* mRNA significantly rose in WT thymus at GD16.5 as compared to NP (*p* = 0.0041), whereas transcripts for *Esr1, Esr2, Pgrmc1, Pgrmc2* remained unchanged (**Figure 2A**, *p* > 0.05). We also conducted immunofluorescence to understand the spatial distribution and dynamics of PGR protein expression throughout gestation. PGR expression was sparse in NP thymus but increased dramatically by GD14.5 (**Figure 2B**, *p* < 0.0001, **Supplementary Figure 2**). Additionally, PGR immunofluorescence using anti-PGR antibody showed nuclear localization in large reticular cells suggestive of thymic epithelial cells (TECs), found mostly in the cortex (**Figure 2C**).

**Figure 3:**
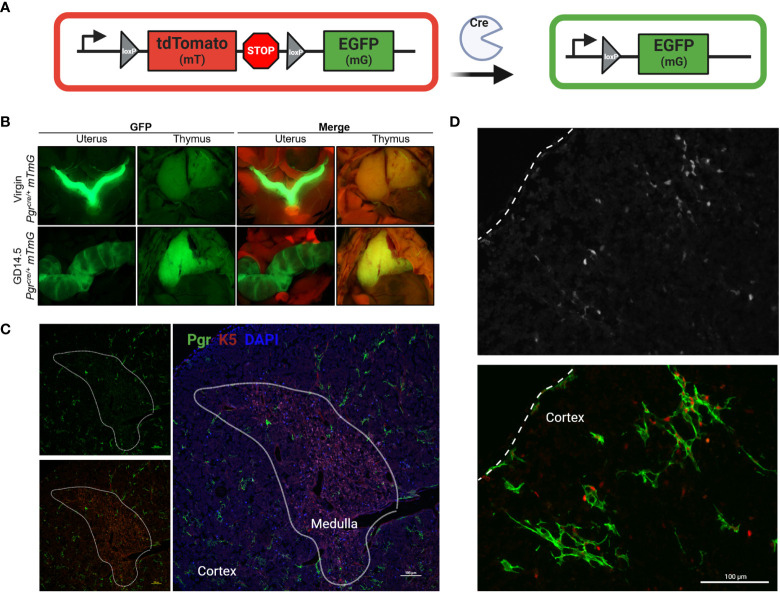
*Pgr* reporter system identifies PGR + cells in the cortex of the thymus in pregnancy. **(A)** Explanation of Cre/Rosa26mTmG reporter system. In the absence of Cre recombinase activity, all cells remain mT+ (red). Upon expression of *Pgr* gene, Cre recombinase generation mediates the excision of the mT cassette. Post recombination, the cell membrane of all PGR+ cells will become GFP+. **(B)** Macroscopic visualization of GFP expression in the thymus at GD14.5 compared to virgin control. **(C)** Partially stitched *Pgr^cre/+^;Rosa26^mTmG/mTmG^* thymus at GD14.5 to demonstrate GFP+ expression in the cortex of the thymus. K5 denotes medulla. DAPI = nuclei, PGR = GFP+ cells. The dotted lines outline the medulla. Magnification: 200x, Scale bar = 100μm. **(D)** *Pgr^cre/+^;Rosa26^mTmG/mTmG^* thymus at GD14.5 stained with anti-PGR primary antibody and counter stained with anti-rabbit secondary antibody (Alexa Fluor 405), pseudo-colored red to provide contrast to GFP+ cells. Dotted line indicates the capsule of the thymus. Magnification: 200x, Scale bar = 100μm.

We confirmed the rise in cellular expression and location of *Pgr* using a dual reporter mouse model, *Pgr^cre/+^;Rosa26^mTmG/+^*, in which cells that have expressed *Pgr* gene are identified by membrane-associated GFP reporter (**Figure 3A**). Macroscopic visualization showed a striking increase in *Pgr*-driven GFP expression by GD14.5 as compared to NP thymus (**Figure 3B**). This increase could also be seen at the cellular level, which confirmed the lack of expression in thymocytes, with GFP reporter expression consistent with *Pgr* expression by thymic epithelial cells (**Figures 3C, D**). No GFP was observed in control *Pgr^+/+^;Rosa26^mTmG/+^* GD14.5 thymi (**Supplementary Figure 3**).

### TEC Specific *Pgr* Deletion Prevents Thymic Involution in Pregnancy

**Figure 4:**
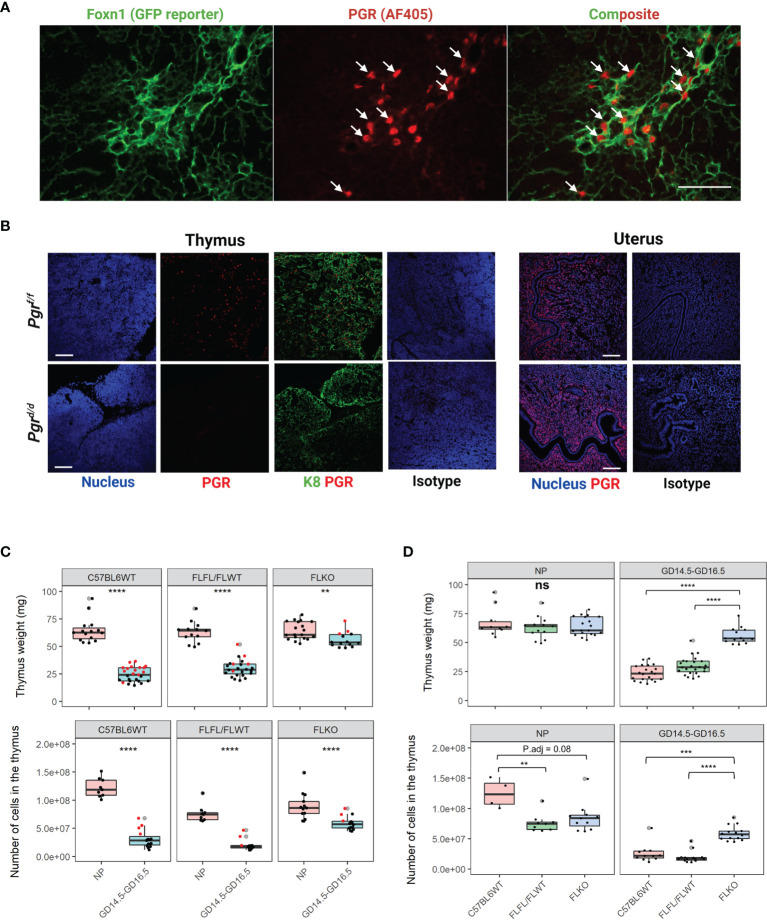
Conditional deletion of *Pgr* gene from the Foxn1+ thymic epithelial cells prevents thymic involution in pregnancy. **(A)** Nuclear progesterone receptor (PGR) colocalizes with Foxn1+ Thymic epithelial cells (TECs) at GD6.5. Alexa flour 405 was used as a secondary antibody. PGR is pseudo-colored red to provide contrast to the GFP+. 400x magnification, scale bar = 50μm. **(B)** Immunofluorescence staining to detect PGR protein confirms the conditional deletion of *Pgr* in the thymus at GD16.5 of *Pgr^d/d^* females. PGR expression is intact in the NP female uterus of both *Pgr^f/f^* controls and *Pgr^d/d^* animals. Magnification at 200x. Scale bar = 100μm, **(C)** Thymic weight (Top) and cellularity (Bottom) in NP and GD16.5 C57BL6WT, *Pgr^f/f^*/*Pgr^f/+^* (FLFL/FLWT) or *Pgr^d/d^* (FLKO) females. Red dots = GD14.5. **(D)** Thymic weight (Top) and cellularity (Bottom) in C57BL6WT, *Pgr^f/f^*/*Pgr^f/+^* (FLFL/FLWT) or *Pgr^d/d^* (FLKO) between NP and pregnant (GD14.5 - GD16.5) females. Each dot represents one mouse. Statistical analysis was performed using non-parametric Wilcoxon test **(C)** or Kruskal-Wallis test followed by Dunn’s test for *post-hoc* assessment with Bonferroni correction **(D)**. P<0.05 deemed significant. **p < 0.001, ***p < 0.0005, ****p < 0.00001, ns, non-significant.

To test the significance of TEC-PGR in pregnancy-associated thymic involution, we created a conditional deletion model in which *Pgr* gene is deleted specifically from TEC. To demonstrate that Foxn1+ TECs expressed PGR, we crossed *Rosa26^mTmG/+^* mice (9) with *Foxn1^cre/cre^* mice, in which Cre expression is driven by the TEC-specific promoter for *Foxn1* (10, 12). Combining this model with PGR immunofluorescence confirmed the expression of PGR predominantly by Foxn1+ TECs in pregnancy (**Figure 4A**).

To ablate *Pgr* expression from Foxn1+ TECs, we crossed *Foxn1^cre/cre^* mice with *Pgr^f/f^* mice, and then backcrossed F1 females to *Pgr^f/f^* males. This cross abolished thymic PGR protein expression in *Pgr*-deficient (*Pgr^d/d^*) mice but not in flox controls (*Pgr^f/f or f/+^*); uterine expression of PGR protein remained unaffected (**Figure 4B**). The serum concentration of estradiol and progesterone were also comparable between controls and *Pgr^d/d^* animals (**Supplementary Figure 4A**). We then established timed mating of *Pgr^f/f^* and *Pgr^f/+^* control and sacrificed animals at GD16.5. In pregnancy, the thymus weight of GD14.5-16.5 WT and floxed control animals both decreased significantly (*p* < 0.0001), by approximately 2.8-fold and 2.1-fold, respectively. Thymi of *Pgr^d/d^* animals also decreased (*p* < 0.001), but to a lesser extent (1.1-fold). Similarly, the cellularity of the thymus dropped dramatically in both WT and floxed control animals during pregnancy (5.6-fold and 5-fold, respectively; *p* < 0.00001). Cellularity of *Pgr^d/d^* females also decreased during pregnancy, but to a lesser extent (1.5-fold) (**Figure 4C**). We also compared the thymic weight between genotypes. While the thymic weight was comparable between genotypes in non-pregnant state, in pregnancy, we found that thymic weight of *Pgr*-deficient females was significantly higher compared to our pregnant controls (**Figure 4D**, *p* < 0.00001). Thymic cellularity corresponded with thymic weight as well. Overall, there was approximately a 5.6-fold change in thymic cellularity between NP and GD14.5-GD16.5 for C57BL6WT, 5-fold change for flox controls, and only 1.5-fold change for our *Pgr-*deficient females. We did not observe significant difference between GD14.5 and GD16.5 thymic cellularity in any of the genotypes (**Supplementary Figure 5**, *p* > 0.05).

To determine whether the thymic architectural changes were also apparent in our transgenic mouse model in pregnancy, we stained the thymus of *Pgr^d/d^* and flox controls at GD16.5 and compared the distribution of the cortex to the medulla (**Supplementary Figure 4B**). Our flox control thymus at GD16.5 resembled that of the wild-type thymus at similar gestation of pregnancy (**Figure 1**). Interestingly, the architecture of the *Pgr^d/d^* thymus at GD16.5 resembled that of a NP wild type, with medullary islands distributed throughout the cortex, instead of coalescing in the middle. This outcome suggests a relationship between thymic involution and changes in thymic architecture with pregnancy. In the absence of thymic involution, the architectural changes do not seem to occur.

**Figure 5:**
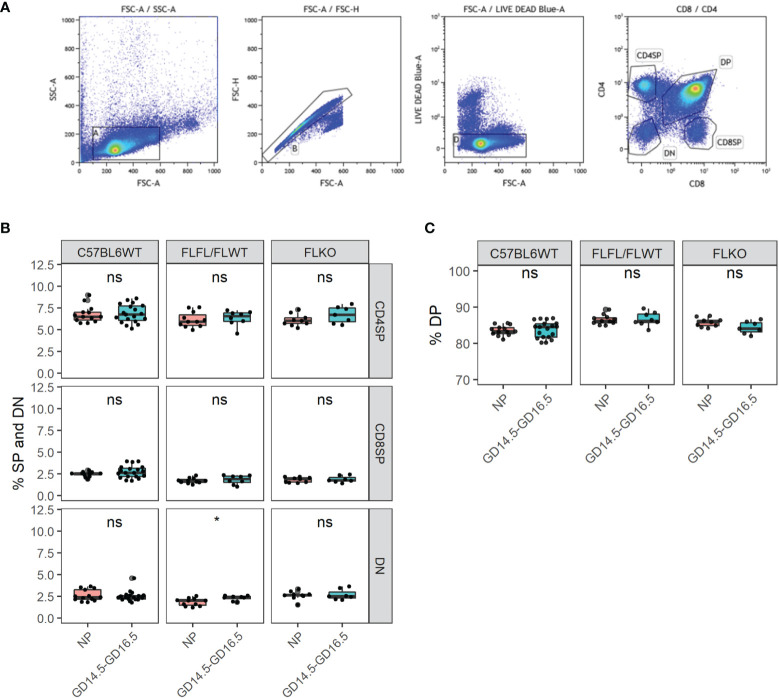
Thymocyte developmental process is undisrupted in the thymus of *Pgr^d/d^* in pregnancy. **(A)** Gating strategy of the murine thymus for CD4SP, CD8SP, DN, and DP. **(B)** % CD4 single positive (SP), CD8SP and DN in WT, FLFL/FLWT and FLKO between NP and GD14.5-GD16.5 of pregnancy. **(C)** % DP population in WT, FLFL/FLWT and FLKO between NP and GD14.5-GD16.5 of pregnancy. Student’s t-test, *p < 0.05, ns, not significant. Each dot represents one mouse.

In order to determine if thymocyte development was impaired in our *Pgr*-deficient females, we conducted flow cytometry from the thymus and measured the proportions of single positive (SP) CD4, CD8 T cells as well as double negative (DN) and double positive (DP) thymocytes from C57BL6WT, flox controls and our *Pgr*-deficient females at NP and pregnant state. Despite the lack of thymic involution in our *Pgr*-deficient females, proportions of CD4, CD8, DN, and DP cells were comparable between NP and late gestation of pregnancy in all genotypes (**Figures 5A–C**, *p* > 0.05). These results demonstrated that the pregnancy-associated thymic involution is dependent on the expression of *Pgr* by Foxn1+ TEC, but the lack of involution does not impact the overall proportions of T cells in the thymus.

### Thymic Epithelial Cell *Pgr* Influences Genes Involved in Self-Antigen Expression and Size of Thymic Medulla

**Figure 6:**
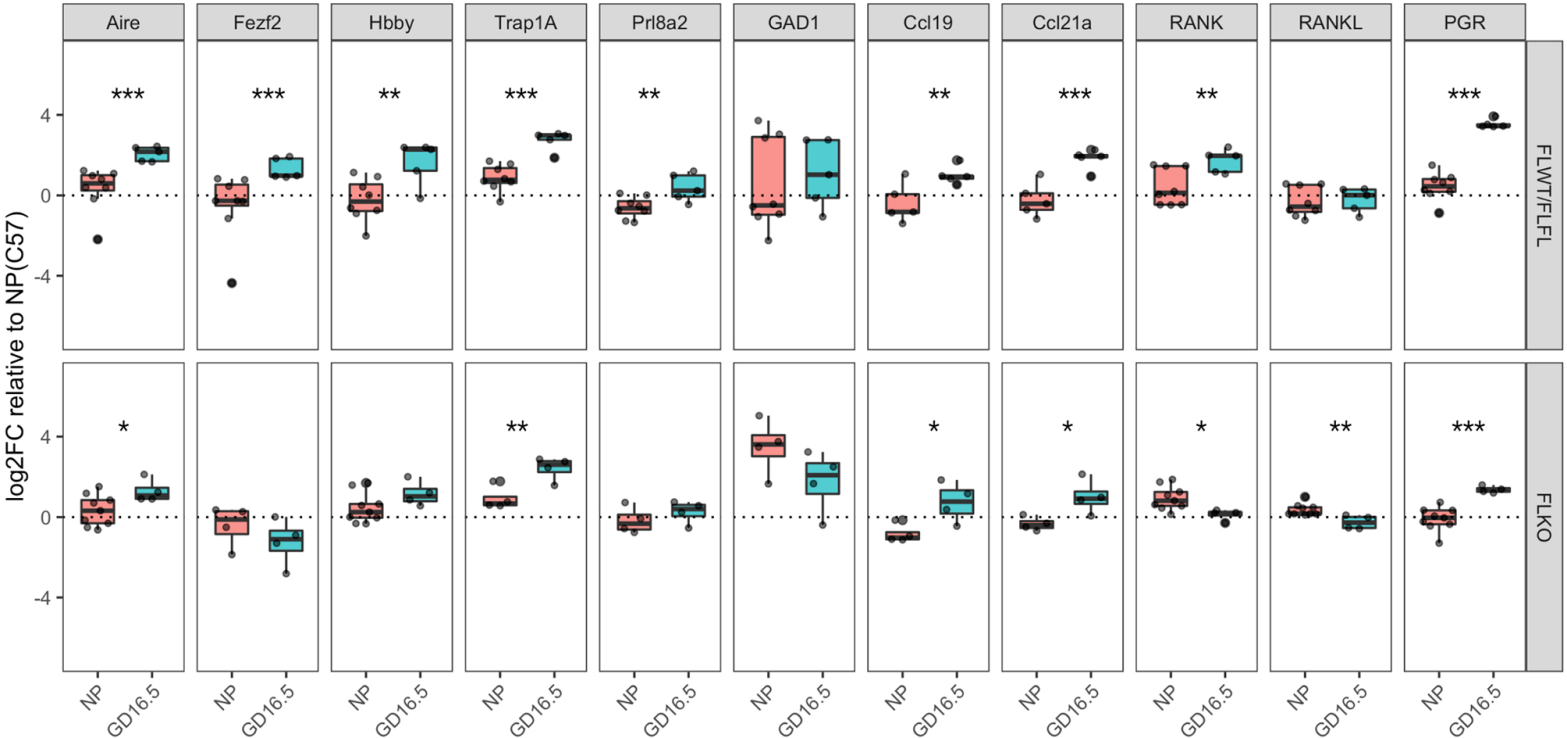
*Pgr^d/d^* thymus show unchanged expression of *Fezf2*, involved in self-antigen expression and decreased expression of *Tnfrsf11a* (RANK), involved in mTEC maintenance. Comparison of gene expression in the thymus of *Pgr^f/+^* or *Pgr^f/f^* (FLWT/FLFL) and *Pgr^d/d^* (FLKO) females between non-pregnant (NP) and GD16.5 of pregnancy. Delta CT was calculated using *Gapdh* as housekeeping gene. Delta CT of each gene from C57BL/6J WT NP thymus samples were used to calculate delta-delta CT, then the values were Log2 transformed to calculate Log2FoldChange (Log2FC). *p < 0.05, **p < 0.01, ***p < 0.001. Each dot represents one mouse.

We next explored whether progesterone regulates expression of genes that could cause changes in thymic function and architecture. We were particularly interested in whether ablation of *Pgr* impacts gene expression by mTECs because Pgr-deficient thymus at pregnant state resembled that of the non-pregnant thymus. Therefore, we compared thymi of *Pgr^f/f^* and *Pgr^d/d^* NP or GD16.5 mice for expression of genes that regulate self-antigens (*Aire, Fezf2*) as well as several tissue-restricted antigens that are found in gestational tissues. We also included *Pgr* to confirm our conditional knock out phenotype, and *Gad1*, an AIRE-independent gene to determine whether its expression changed with pregnancy. In our flox controls, both *Aire* and *Fezf2* increased significantly, which appeared to be partially (*Aire*) and completely (*Fezf2*) dependent on progesterone (**Figure 6**, *p* < 0.001). In addition, genes regulated by *Aire*, including the fetal/placental genes *Hbby (p < 0.01)* and *Trap1a*, (*p* < 0.001) and the decidua-associated gene *Prl8a2 (p < 0.01)*, were all significantly upregulated during pregnancy; this effect was ablated for *Hbby* and *Prl8a2* in pregnant *Pgr^d/d^* mice (**Figure 6**, *p* > 0.05). Finally, genes that regulate thymocyte trafficking (*Ccl19, Ccl21a*) and medullary size (*Tnfsf11/RANKL, Tnfrsf11a/RANK*) were investigated. In our controls, *Ccl19 (p < 0.01), Ccl21a (p < 0.001)*, and *Tnfrsf11a (p < 0.01)* mRNA rose significantly during pregnancy, while *Tnrsf11* mRNA remained unchanged (**Figure 6**). These changes were not affected in *Pgr^d/d^* females, however, mRNA for *Tnfrsf11a (p < 0.05)* and *Tnfrsf11* (*p* < 0.01) significantly dropped in these mice between NP and GD16.5 of pregnancy.

### Fertility Trial

**Figure 7:**
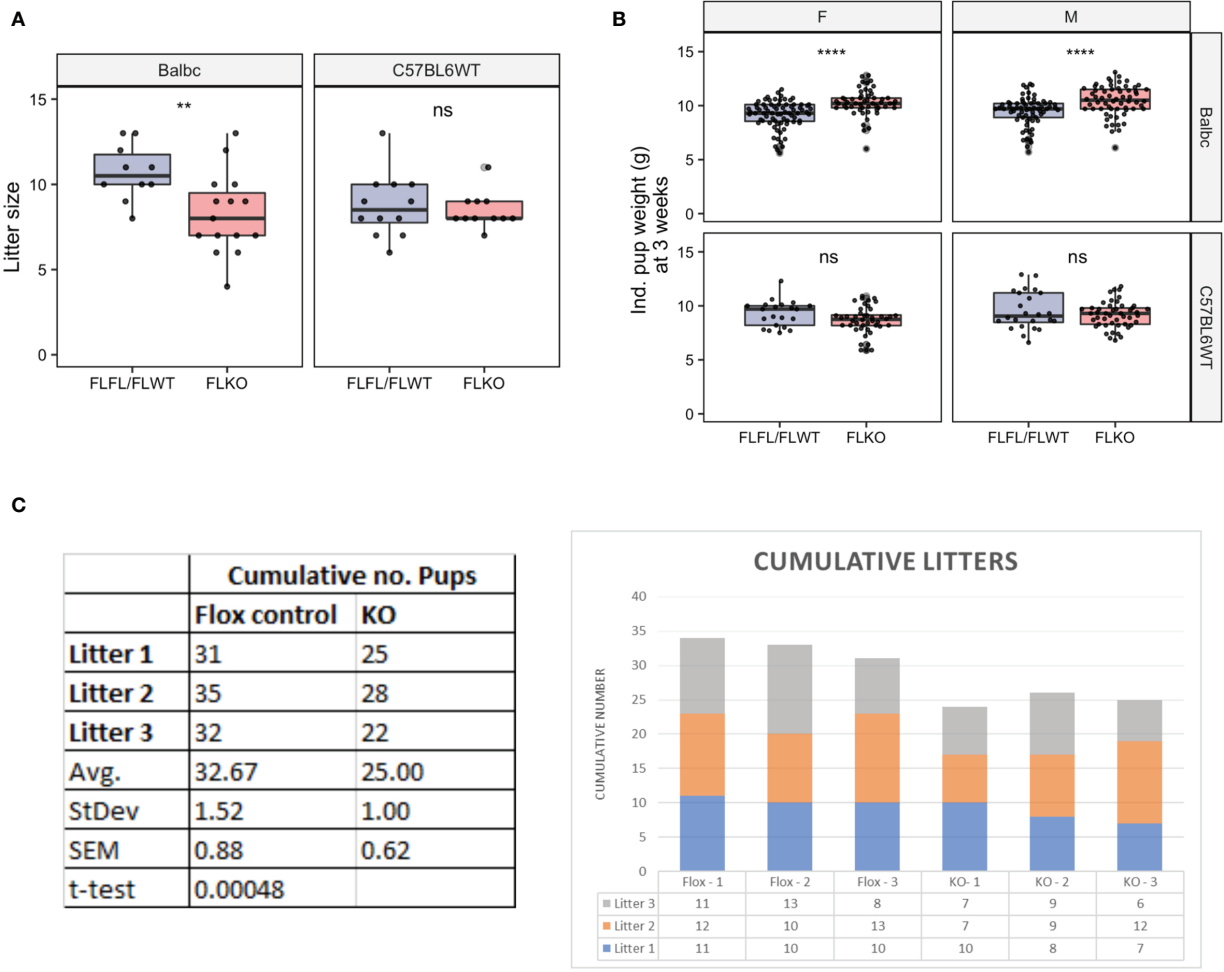
Assessment of long-term fertility outcome in FLFL/FLWT (*Pgr^f/f^* or *Pgr^f/+^*) and FLKO (*Pgr^d/d^*) in allogeneic and syngeneic mating strategy. **(A)** Individual litter size from female of FLFL/FLWT and FLKO mated with either allogeneic (Balb/c) or syngeneic (C57) male. **(B)** Weight of male or female 3 week old weanlings born to FLFL/FLWT and FLKO from allogeneic or syngeneic mating. **p < 0.01, ****p < 0.0001, ns, not-significant. **(C)** Cumulative numbers of pups across three allogeneic pregnancies in controls (Flox-1 to Flox- 3) and FLKO animals (KO-1 to KO-3).

Previous work showed impaired pregnancy when thymectomized female mice were transplanted with a thymus lacking *Pgr* (5). We asked whether pregnancy is similarly impaired in our TEC-specific *Pgr* conditional KO model. We saw that the mean litter size across syngeneic pregnancies was slightly diminished at GD16.5 in our *Pgr^d/d^* animals; however, the difference was not statistically significant [**Supplementary Figure 6**, 8.42 ± 1.60 (P*gr^f/f^* and *Pgr^f/wt^*) VS. 7.54 ± 2.62 (*Pgr^d/d^*), *p* = 0.81]. Similarly, the proportion of resorbed fetuses were not different between controls (9.32%, or 11/118 implantation sites, of 14 control dams) and *Pgr^d/d^* (14.45%, or 12/83 implantation sites, of 11 *Pgr^d/d^* dams, *p*>0.05, chi-square analysis). We also assessed overall fertility by pairing control or *Pgr^d/d^* females with either C57BL/6 males (syngeneic pregnancy) or Balb/c males (allogeneic pregnancy) for long term fertility trial. The average litter size from *Pgr^d/d^* dams was significantly smaller than that of control dams when mated to allogeneic, but not to syngeneic sires (**Figure 7A**). Correspondingly, the mean pup weight at weaning was higher in these pregnancies; this was true regardless of sex (**Figure 7B**). In addition, cumulative numbers of pups across three pregnancies were smaller in these pregnancies (Controls, 32.76 ± 0.88 vs. 25 ± 1 pups; n=3 dams; *p* < 0.005) (**Figure 7C**). Collectively, the data indicate that fertility in allogeneically-bred *Pgr^d/d^* females is reduced in comparison to allogeneically-bred control females.

## Discussion

The thymus is a primary lymphoid organ that is responsible for the generation of a self-tolerant T cell repertoire (13). While the thymus involutes irreversibly with age in both sexes (14), the female thymus undergoes a dramatic but reversible hormone-mediated structural involution during pregnancy. In this study, we sought to understand the role and functional significance of progesterone-mediated thymic involution during pregnancy. We found that PGR is expressed primarily in cortical thymic epithelial cells (cTECs), and that its expression is strongly and specifically upregulated during pregnancy. Further, we showed that pregnancy-induced thymic involution is driven by expression of *Pgr* by Foxn1+ TECs, with other, as-yet-undetermined *Pgr*-independent mechanisms also in play. Targeted deletion of *Pgr* in these cells minimized pregnancy-induced thymic involution and disrupted pregnancy-induced changes in expression of several genes that may be important to thymic function during pregnancy. The lack of thymic involution, however, did not impact the proportions of single positive CD4, CD8 T cells, double negative or double positive thymocytes cells between NP and pregnant transgenic animals. Finally, absence of *Pgr* in cTEC did not affect outcomes of syngeneic pregnancy but compromised multiparous allogeneic pregnancy.

Most notably, thymic involution during pregnancy was associated with “coalescing” of the medullary area in the WT thymus. Therefore, we investigated whether medulla specific genes changed during pregnancy. In WT females, pregnancy is associated with changes in expression of genes important for mTEC function, including *Aire, Fezf2*, and several tissue-specific genes that are specific to the fetal and maternal tissues at the maternal-fetal interface. Aire-dependent gene expression in the maternal thymus could lead to either deletion of CD4 and CD8+ T cells that are specific to these antigens, development of thymic regulatory T cells, or both. Either outcome could ensure maternal immune tolerance to antigens specific to the maternal-fetal interface during pregnancy. Consistent with this idea, it was recently shown that Aire may play a role in maternal immune tolerance to the fetus and early implantation events (15, 16). The increase in *Aire* mRNA expression at GD16.5 compared to NP correlates with increase in Aire-dependent self-antigen expression including *Hbby*, *Prl8a2* and *Trap1a*. *Hbby* encodes fetal hemoglobin, and *Trap1a* is a cancer antigen that is strongly expressed in the placenta with a possible role in cell migration, but unknown function in the placenta (17, 18). *Prl8a2* is specifically expressed by the endometrial decidual cells (19–21). This intriguing finding is the first to demonstrate that the maternal thymus expresses genes specific to the fetus, placenta, and endometrium during pregnancy. Interestingly, the expression of these genes also significantly increased in *Pgr^d/d^* thymus between NP and GD16.5, indicating that the lack of PGR does not influence the expression of *Aire* and *Aire*-dependent genes in pregnancy, despite the lack of architectural changes in pregnancy.

Conversely, we observed decreased expression of *Tnfrsf11a* (RANK) and *Tnfsf11* (RANKL) between NP and GD16.5 in *Pgr^d/d^* thymus. This is interesting because RANK is involved in the development of mTECs (22). We postulated that RANK expression may be increased in the thymus in pregnancy due to the cortical-to-medullary changes that occur during this time. Indeed, we observed increase in *Tnfrsf11a* mRNA expression in WT between NP and GD16.5; however, *Tnfrsf11a* mRNA expression decreased between NP and GD16.5 in *Pgr^d/d^* thymus. This decreased expression of *Tnfrsf11a* mRNA in *Pgr^d/d^* could explain the thymic architecture of *Pgr^d/d^* at late gestation of pregnancy.

Unexpectedly, we observed no change in *Fezf2* mRNA expression in *Pgr^d/d^*. Like Aire, which is a nuclear transcription factor expressed by mTECs to regulate expression of tissue specific antigens (23), Fezf2 regulates transcription of tissue-restricted antigens similarly, but not overlapping, with those regulated by Aire (24). Expression of Aire by mTECs and subsequent expression of tissue-restricted antigens mediate the differentiation of naïve CD4 T cells into regulatory T cells, which are especially unique due to their expression of self-antigen specific T cell receptors (25). Because Fezf2 regulates expression of self-antigens in the thymus (24), its expression may complement Aire in the generation of a self-tolerant T cell repertoire during pregnancy. Since the expression of *Fezf2* remains unchanged in *Pgr^d/d^* thymus at late gestation, unlike our flox controls, this may impact the repertoire of thymic regulatory T cells generated in the presence or absence of *Pgr* expression. Further research is required to test whether this is true.

Tibbetts et al. (5) was the first to suggest that the expression of PGR by TECs influenced thymic involution, and to demonstrate that thymic involution in pregnancy was necessary for optimal pregnancy outcome. We elaborate on the findings of Tibbetts et al. (5) by specifically ablating *Pgr* in Foxn1+ TECs and pinpointing the expression to TECs in the cortex. We conducted fertility trial to validate the findings of Tibbetts and colleagues. Our long-term fertility trial showed that Pgr-deficient dams carried significantly smaller litter size compared to our flox controls. However, in comparison to the results of Tibbetts et al., we observed 14% resorption of embryos compared to 40% when checked at GD16.5. We suspect that the external stress imposed on the dams from embryo transfer and surgery conducted by Tibbetts group may have resulted in increased resorption rate. For future studies, we can directly test whether our conditional knockout model is also susceptible to stress-induced pregnancy loss to address this discrepancy.

We cannot exclude the potential of progesterone acting on thymocytes through the membrane progesterone receptors to mediate the effect of progesterone through non-genomic route to reduce proliferation of thymocytes, leading to subsequent involution of the thymus in murine pregnancy. In bovine CD4+ T cells obtained from corpus luteum (and peripheral blood), membrane progesterone receptor, PAQR7, is clearly expressed on the cell membrane, to which progesterone binds to decrease phosphorylation of Zap70, with a subsequent decrease in T cell receptor activation (26). Additionally, glucocorticoid receptor (GR) and PGR recognize the same DNA binding elements as their DNA binding domain share 90% amino acid sequence (27, 28). Progesterone can interact with GR expressed by the thymocytes and induce differentiation to regulatory T cells in the thymus (29). Therefore, other signaling pathways of progesterone interaction cannot be excluded and warrant further investigation.

In summary, we show for the first time that the PGR is expressed by Foxn1+ thymic epithelial cells, and its expression increases dramatically during pregnancy, particularly in cTECs. Pregnancy-associated increases in hormone receptors are specific to PGR, as neither estrogen receptors nor membrane-associated progesterone receptors changed in pregnancy. Consistent with this observation, we show that loss of thymic weight and cellularity is at least partially dependent on TEC-PGR, as TEC-specific deletion of the receptor shortened pregnancy-induced architectural and phenotypic changes. PGR expression in TEC appears to influence fertility in allogeneic pregnancies, as well as expression of genes that may influence maternal tolerance of the fetus. These results reveal and confirm the importance of hormone-mediated effects on the maternal thymus during pregnancy. Future studies will further elucidate the effects of progesterone on changes in T cell populations produced by the thymus, and identify genes regulated by PGR in cTEC by conducting RNAseq and ChIP-seq experiments.

## Data Availability Statement

The original contributions presented in the study are included in the article/**Supplementary Material**. Further inquiries can be directed to the corresponding author.

## Ethics Statement

The animal study was reviewed and approved by Institutional Animal Care and Use Committee at Michigan State University.

## Author Contributions

SA and MP designed the study. SA performed the experiments, conducted analysis and wrote the manuscript. SN assisted with data analysis using RStudio. JL provided PGR flox animals. MP provided institutional support, guidance and resources. RA assisted with data analysis using IMARIS. T-HK and J-WJ provided PGRcre animals. All authors contributed to and approved of the manuscript content.

## Funding

Supported by NIH grants AI143173 and HD100832 (PGP). The University of Virginia Center for Research in Reproduction Ligand Assay and Analysis Core is supported by Eunice Kennedy Shriver NICHD/NIH Grant R24HD102061.

## Conflict of Interest

The authors declare that the research was conducted in the absence of any commercial or financial relationships that could be construed as a potential conflict of interest.

The reviewer S-PW declared a past collaboration with two of the authors JL and J-WJ to the handling editor.

## Publisher’s Note

All claims expressed in this article are solely those of the authors and do not necessarily represent those of their affiliated organizations, or those of the publisher, the editors and the reviewers. Any product that may be evaluated in this article, or claim that may be made by its manufacturer, is not guaranteed or endorsed by the publisher.

## Supplementary Material

Supplementary Figure 1 Example of PCR genotyping results.

Supplementary Figure 2 Representative immunoflurescence images taken at 200x magnification of the thymus used to calculate percentages represented in **Figure 2B**. Scale bar = 100um.

Supplementary Figure 3 Thymus was excised and sectioned at 5 μm from *Pgr^WT^;Rosa^mTmG/mTmG^* female at GD14.5 to demonstrate that *Pgr^Cre^* activity in the *Rosa^mTmG/mTmG^* reporter females during pregnancy is specific. Scale bar = 2000 μm. Magnification = 100x.

Supplementary Figure 4
**(A)** Serum progesterone (P4, Left) and estradiol (E2, Right) levels in non-pregnant (NP) and GD16.5 dams from control C57BL/6 females compared to *Pgr^f/+^* and *Pgr^f/f^* (FLWT/FLFL) and *Pgr^d/d^* (FLKO) females. n=3 for all conditions and genotype. Statistical analysis were performed using student’s t-test between NP and GD16.5 for each genotype. *p< 0.05; **p < 0.01, ***p<0.001, ns = non significant. **(B)** GD16.5 thymus of *Pgr^d/d^* (left) and *Pgr^f/f^* (right) dual stained with anti-cytokeratin 5 (K5) and anti-cytokeratin 8 (K8) to characterize medullary islands (K5) in the field of cortex (K8).

Supplementary Figure 5
**(A)** Thymus weight in C57BL6WT, *Pgr^f/+^* and *Pgr^f/f^* (FLWT/FLFL) or *Pgr^d/d^* (FLKO). **(B)** Number of cells in the thymus of C57BL6WT, *Pgr^f/+^* and *Pgr^f/f^* (FLWT/FLFL) or *Pgr^d/d^* (FLKO) thymus. Statistical analysis was performed using non-parametric Kruskal-Wallis test with post doc Dunn test. P-values adjusted using Bonferroni method. *p<0.05, **p<0.01, ****p<0.00005, ns = non significant.

Supplementary Figure 6 Assessment of short-term fertility outcome in *Pgr^f/f^* or *Pgr^f/+^* and *Pgr^d/d^* in syngeneic mating strategy. **(A)** Size of the litter born from each genotype mated with C57BL6/J males for syngeneic mating. Each point represents a dam. **(B)** Average weight of pups at GD16.5 in each genotype. Each point represents a dam. **(C)** Calculation of % resorbed fetus at GD16.5 by comparing number of live pups to resorbed sites in each genotype.
