# Corrigendum: Nuclear Progesterone Receptor Expressed by the Cortical Thymic Epithelial Cells Dictates Thymus Involution in Murine Pregnancy

**DOI:** 10.3389/fendo.2022.958735

**Published:** 2022-06-21

**Authors:** Soo Hyun Ahn, Sean L. Nguyen, Tae Hoon Kim, Jae-Wook Jeong, Ripla Arora, John P. Lydon, Margaret G. Petroff

**Affiliations:** ^1^ Department of Pathobiology Diagnostic Investigation, College of Veterinary Medicine, Michigan State University, East Lansing, MI, United States; ^2^ Institute for Integrative Toxicology, Michigan State University, East Lansing, MI, United States; ^3^ Cell and Molecular Biology Program, Michigan State University, East Lansing, MI, United States; ^4^ Department of Obstetrics, Gynecology & Reproductive Biology, Michigan State University, Grand Rapids, MI, United States; ^5^ Department of Obstetrics, Gynecology, and Reproductive Biology, Institute for Quantitative Health Science and Engineering, Michigan State University, East Lansing, MI, United States; ^6^ Department of Molecular and Cellular Biology, Baylor College of Medicine, Houston, TX, United States; ^7^ Department of Microbiology and Molecular Genetics, Michigan State University, East Lansing, MI, United States

**Keywords:** thymus, pregnancy, involution, fertility, progesterone receptor (PGR)

In the published article, there was an error in the legend for Figure 5. The first sentence of the legend incorrectly stated that thymocyte development is “dysfunctional”. It should have stated that thymocyte development is “undisrupted”. The corrected legend appears below.

“Thymocyte developmental process is undisrupted in the thymus of *Pgr^d/d^* in pregnancy. (A) Gating strategy of the murine thymus for CD4SP, CD8SP, DN, and DP. (B) % CD4 single positive (SP), CD8SP and DN in WT, FLFL/FLWT and FLKO between NP and GD14.5-GD16.5 of pregnancy. (C) % DP population in WT, FLFL/FLWT and FLKO between NP and GD14.5-GD16.5 of pregnancy. Student’s t-test, *p < 0.05, ns, not significant. Each dot represents one mouse.”

The authors apologize for this error and state that this does not change the scientific conclusions of the article in any way. The original article has been updated.

## Publisher’s Note

All claims expressed in this article are solely those of the authors and do not necessarily represent those of their affiliated organizations, or those of the publisher, the editors and the reviewers. Any product that may be evaluated in this article, or claim that may be made by its manufacturer, is not guaranteed or endorsed by the publisher.

