# Supplementary figures and images for: Nuclear Progesterone Receptor Expressed by the Cortical Thymic Epithelial Cells Dictates Thymus Involution in Murine Pregnancy
Source: Front Endocrinol (Lausanne). 2022 Apr 14;13:846226. doi: 10.3389/fendo.2022.846226 (PMC9046655; doi:10.3389/fendo.2022.846226)

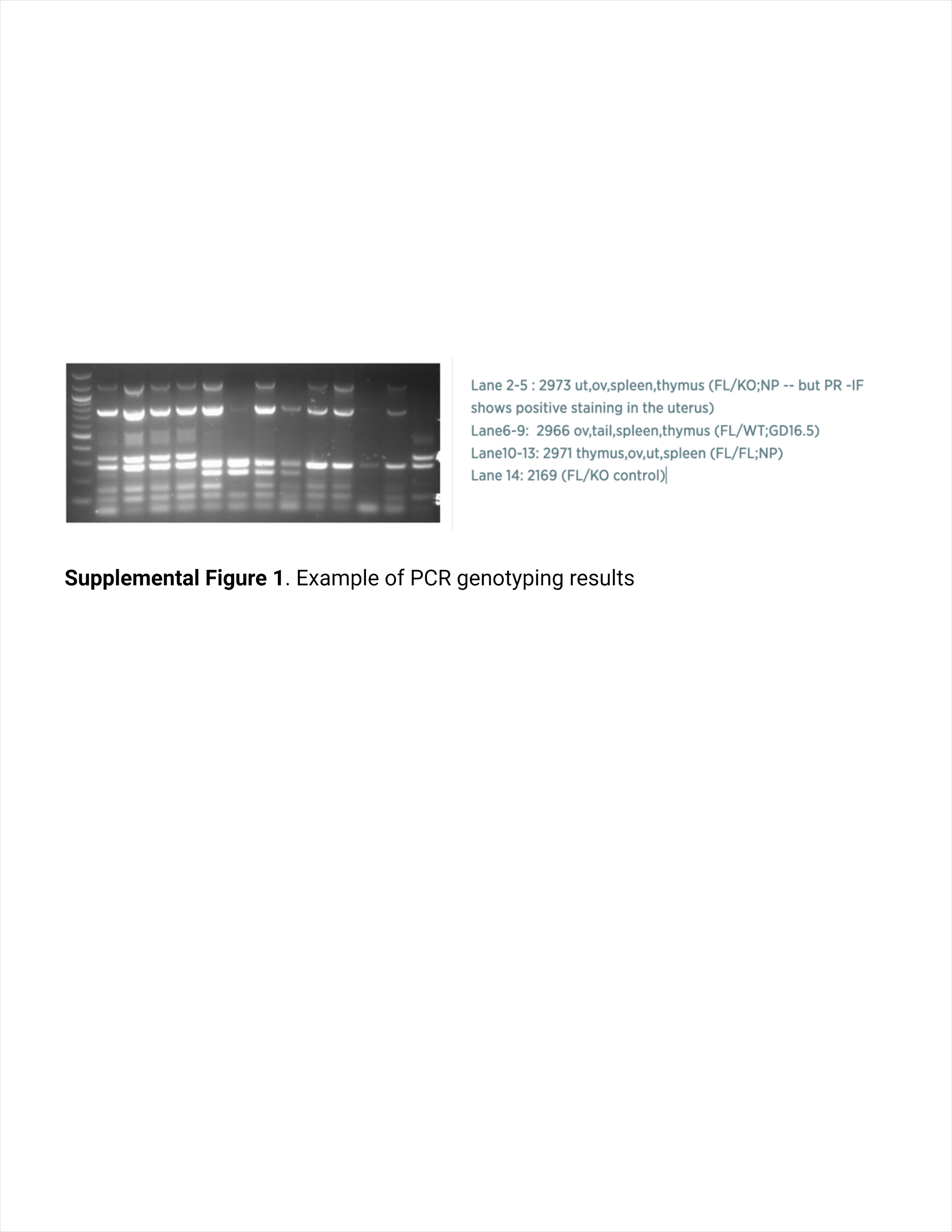

Supplement: Supplementary Figure 1 — Example of PCR genotyping results. [file Image_1.jpeg]

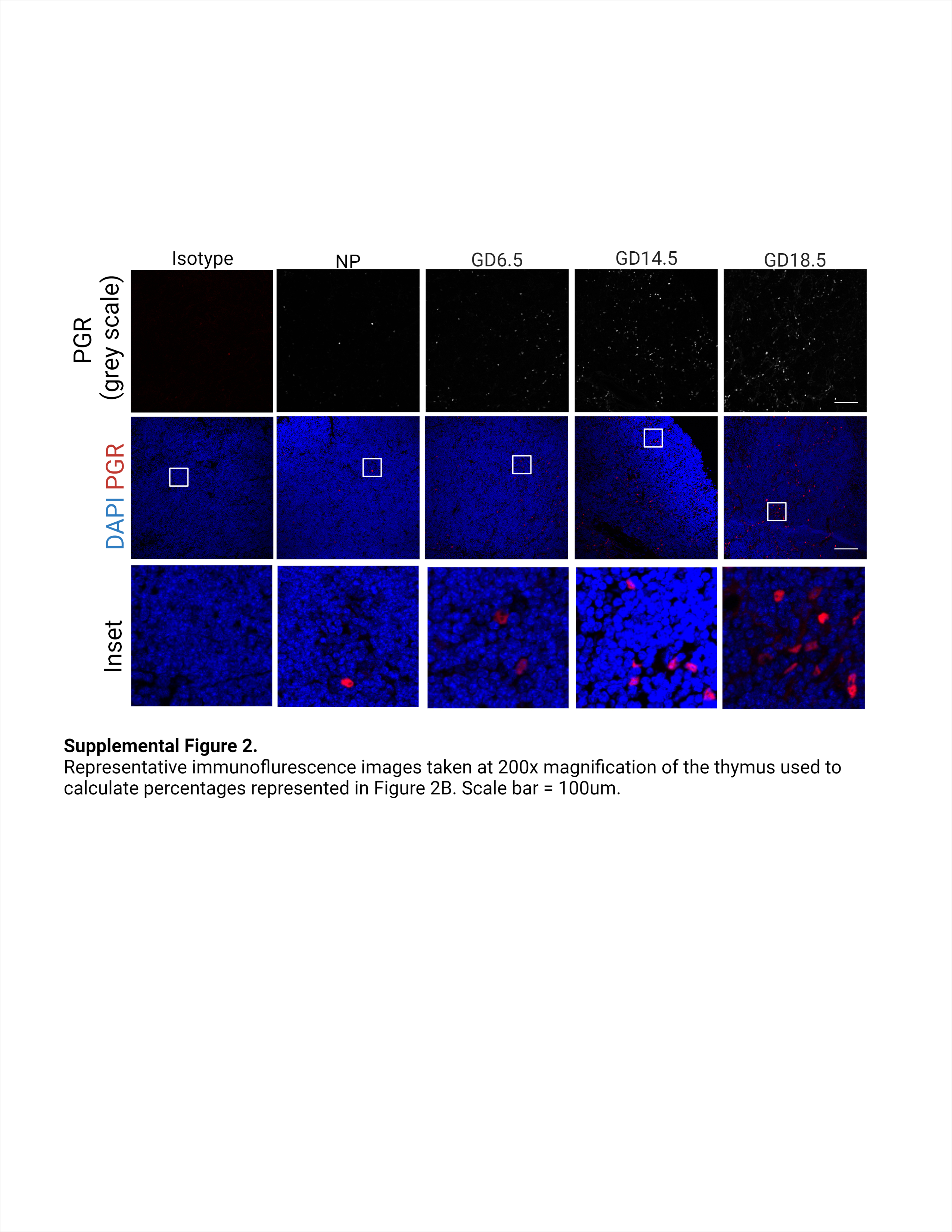

Supplement: Supplementary Figure 2 — Representative immunoflurescence images taken at 200x magnification of the thymus used to calculate percentages represented in Figure 2B . Scale bar = 100um. [file Image_2.jpeg]

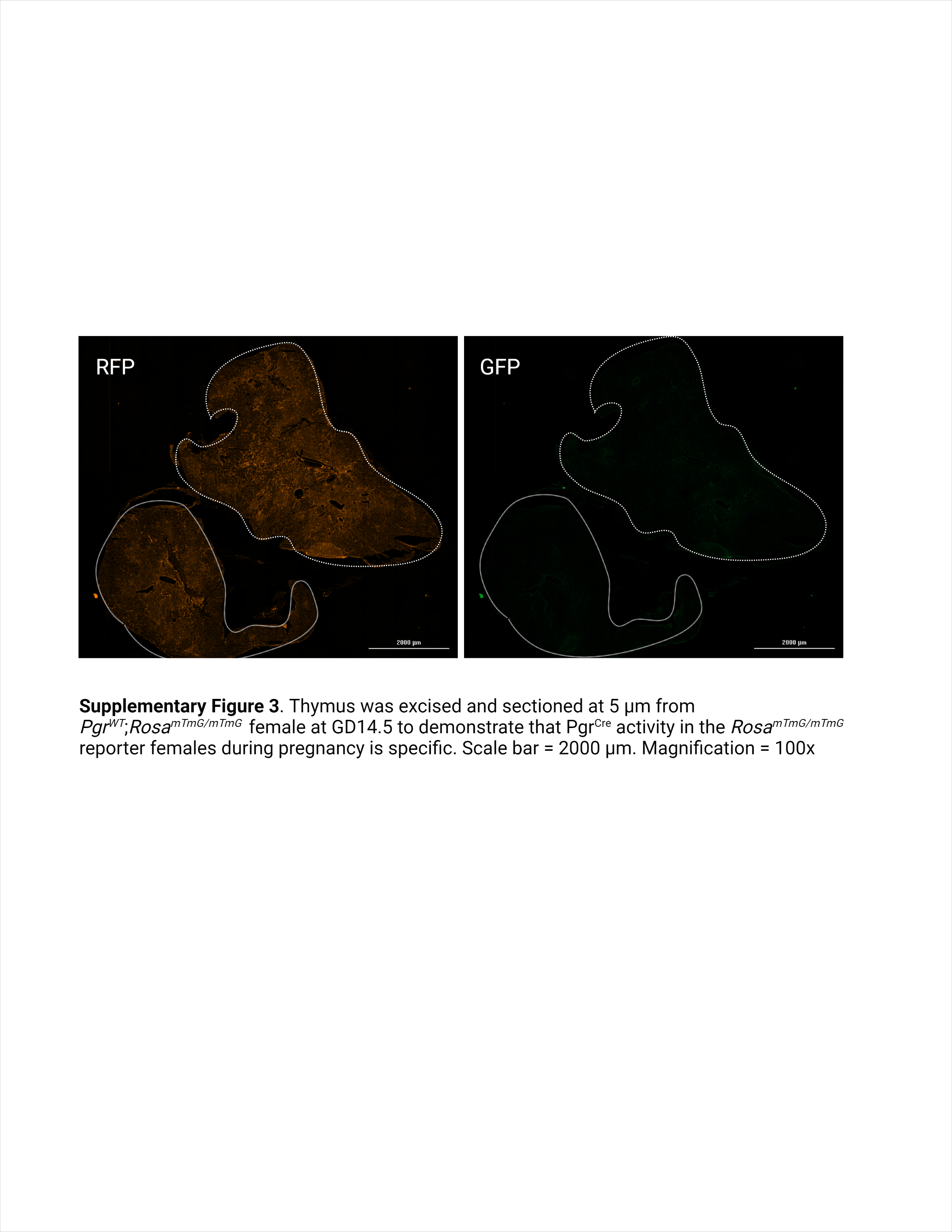

Supplement: Supplementary Figure 3 — Thymus was excised and sectioned at 5 μm from PgrWT;RosamTmG/mTmG female at GD14.5 to demonstrate that PgrCre activity in the RosamTmG/mTmG reporter females during pregnancy is specific. Scale bar = 2000 μm. Magnification = 100x. [file Image_3.jpeg]

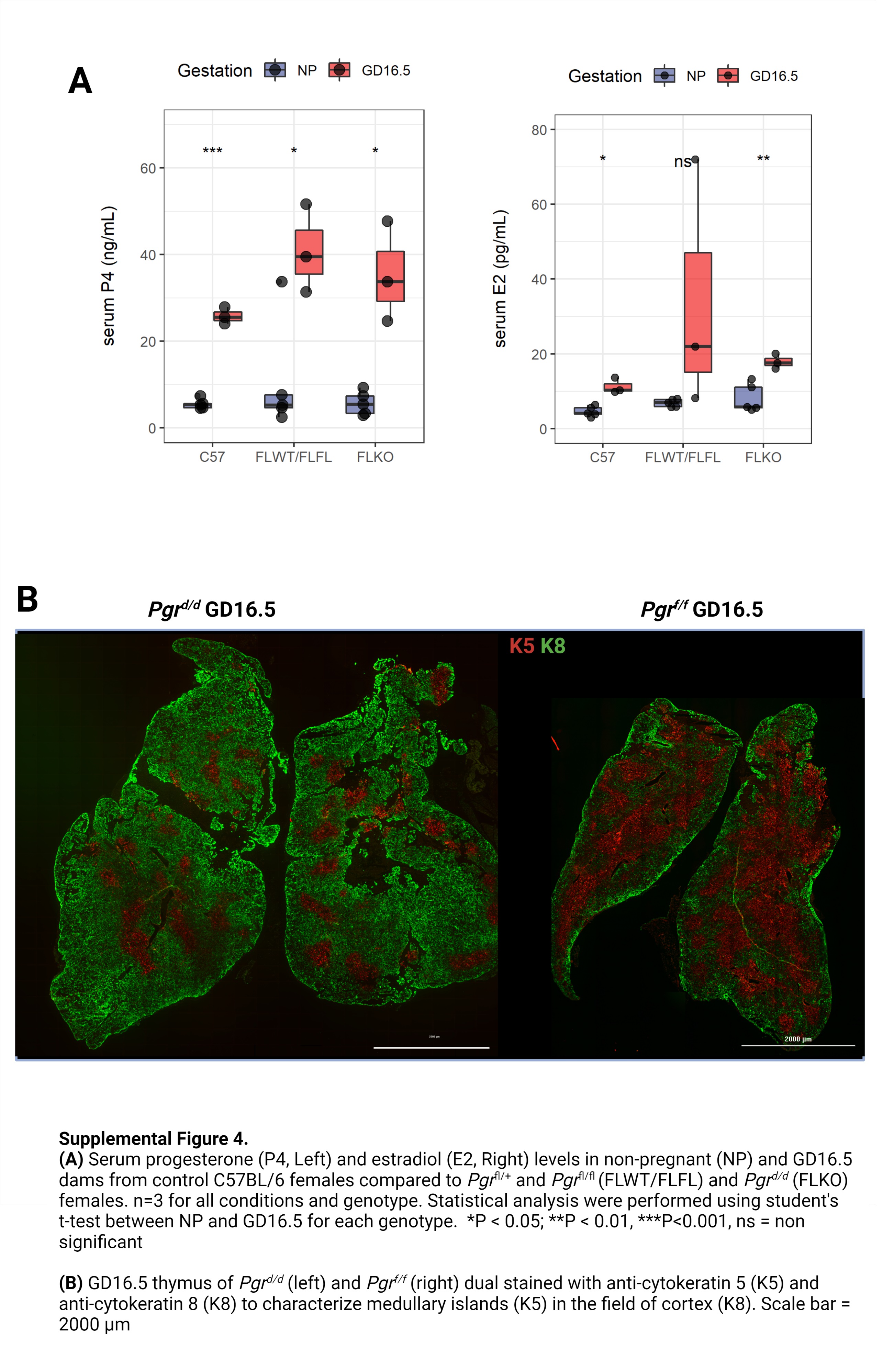

Supplement: Supplementary Figure 4 — (A) Serum progesterone (P4, Left) and estradiol (E2, Right) levels in non-pregnant (NP) and GD16.5 dams from control C57BL/6 females compared to Pgrf/+ and Pgrf/f (FLWT/FLFL) and Pgrd/d (FLKO) females. n=3 for all conditions and genotype. Statistical analysis were performed using student’s t-test between NP and GD16.5 for each genotype. *p< 0.05; **p < 0.01, ***p<0.001, ns = non significant. (B) GD16.5 thymus of Pgrd/d (left) and Pgrf/f (right) dual stained with anti-cytokeratin 5 (K5) and anti-cytokeratin 8 (K8) to characterize medullary islands (K5) in the field of cortex (K8). [file Image_4.jpeg]

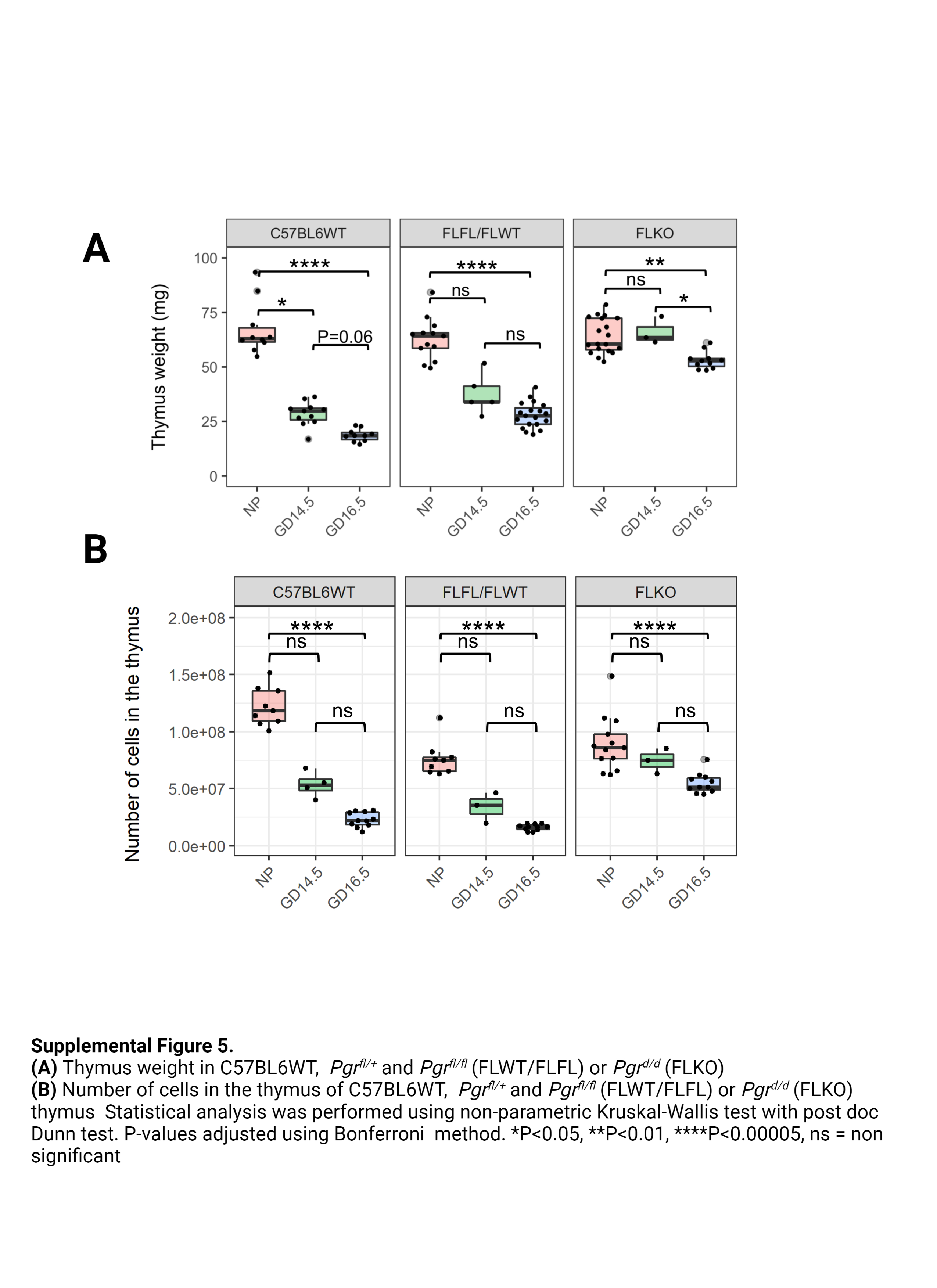

Supplement: Supplementary Figure 5 — (A) Thymus weight in C57BL6WT, Pgrf/+ and Pgrf/f (FLWT/FLFL) or Pgrd/d (FLKO). (B) Number of cells in the thymus of C57BL6WT, Pgrf/+ and Pgrf/f (FLWT/FLFL) or Pgrd/d (FLKO) thymus. Statistical analysis was performed using non-parametric Kruskal-Wallis test with post doc Dunn test. P-values adjusted using Bonferroni method. *p<0.05, **p<0.01, ****p<0.00005, ns = non significant. [file Image_5.jpeg]

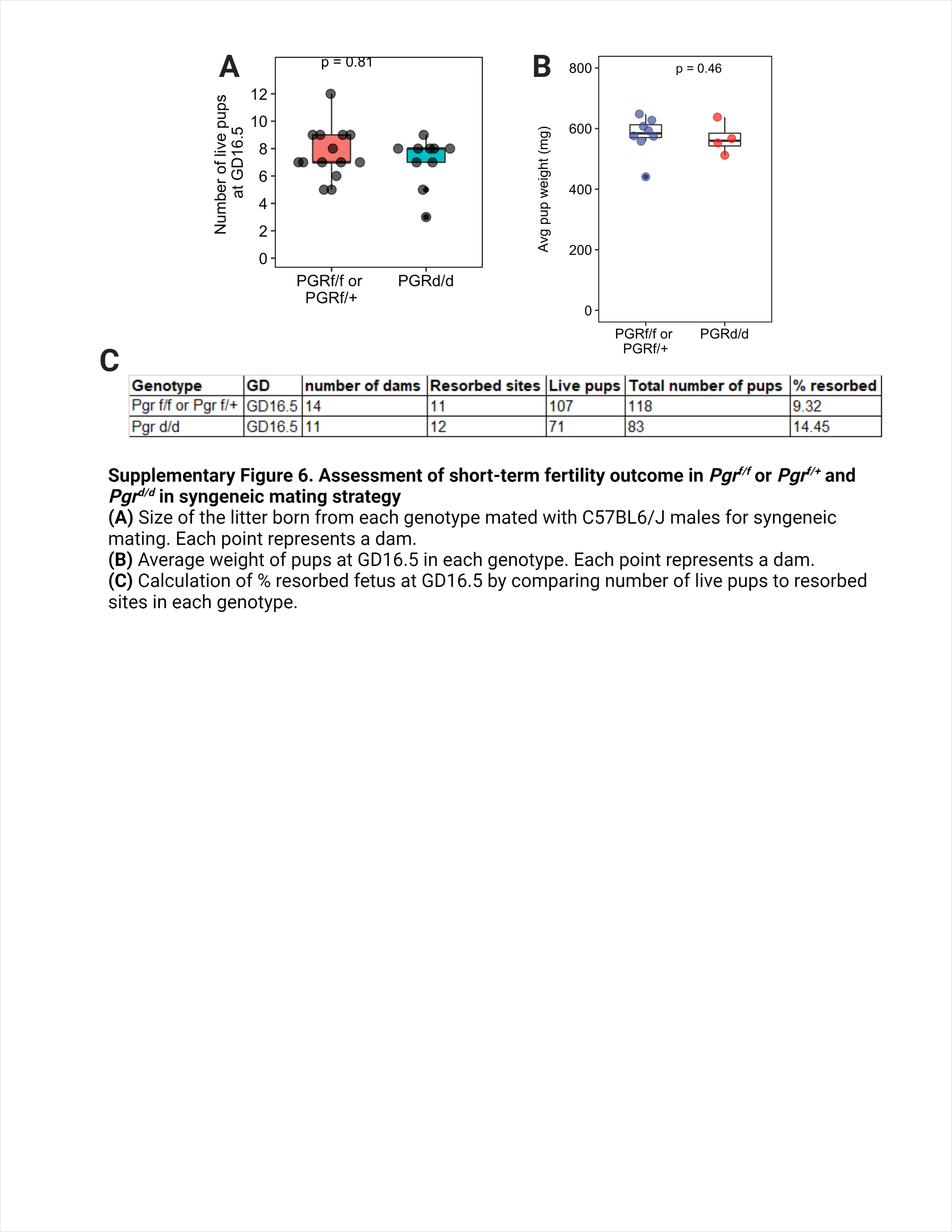

Supplement: Supplementary Figure 6 — Assessment of short-term fertility outcome in Pgrf/f or Pgrf/+ and Pgrd/d in syngeneic mating strategy. (A) Size of the litter born from each genotype mated with C57BL6/J males for syngeneic mating. Each point represents a dam. (B) Average weight of pups at GD16.5 in each genotype. Each point represents a dam. (C) Calculation of % resorbed fetus at GD16.5 by comparing number of live pups to resorbed sites in each genotype. [file Image_6.jpeg]
